# Response of religious groups to HIV/AIDS as a sexually transmitted infection in Trinidad

**DOI:** 10.1186/1471-2458-5-121

**Published:** 2005-11-16

**Authors:** Gillian L Genrich, Brader A Brathwaite

**Affiliations:** 1 Fulbright Fellowship Program for U.S. Students, Port of Spain, Trinidad, West Indies; 2 NCID/DVRD/IDPA, Centers for Disease Control and Prevention, Atlanta, GA, 30333, USA; 3 Center for Medical Sciences Education, Faculty of Medical Sciences, UWI, St. Augustine, Trinidad, West Indies

## Abstract

**Background:**

HIV/AIDS-related stigma and discrimination are significant determinants of HIV transmission in the Caribbean island nation of Trinidad and Tobago (T&T), where the adult HIV/AIDS prevalence is 2.5%. T&T is a spiritually-aware society and over 104 religious groups are represented. This religious diversity creates a complex social environment for the transmission of a sexually transmitted infection like HIV/AIDS. Religious leaders are esteemed in T&T's society and may use their position and frequent interactions with the public to promote HIV/AIDS awareness, fight stigma and discrimination, and exercise compassion for people living with HIV/AIDS (PWHA). Some religious groups have initiated HIV/AIDS education programs within their membership, but previous studies suggest that HIV/AIDS remains a stigmatized infection in many religious organizations. The present study investigates how the perception of HIV/AIDS as a sexually transmitted infection impacts religious representatives' incentives to respond to HIV/AIDS in their congregations and communities. In correlation, the study explores how the experiences of PWHA in religious gatherings impact healing and coping with HIV/AIDS.

**Methods:**

Between November 2002 and April 2003, in-depth interviews were conducted with 11 religious representatives from 10 Christian, Hindu and Muslim denominations. The majority of respondents were leaders of religious services, while two were active congregation members. Religious groups were selected based upon the methods of Brathwaite. Briefly, 26 religious groups with the largest followings according to 2000 census data were identified in Trinidad and Tobago. From this original list, 10 religious groups in Northwest Trinidad were selected to comprise a representative sample of the island's main denominations. In-depth interviews with PWHA were conducted during the same study period, 2002–2003. Four individuals were selected from a care and support group located in Port of Spain based upon their perceived willingness to discuss religious affiliation and describe how living with a terminal infection has affected their spiritual lives. The interviewer, a United States Fulbright Scholar, explained the nature and purpose of the study to all participants. Relevant ethical procedures associated with the collection of interview data were adopted: interviews were conducted in a non-coercive manner and confidentiality was assured. All participants provided verbal consent, and agreed to be interviewed without financial or other incentive. Ethics approval was granted on behalf of the Caribbean Conference of Churches Ethics Committee. Interview questions followed a guideline, and employed an open-ended format to facilitate discussion. All interviews were recorded and transcribed by the interviewer.

**Results:**

Religious representatives' opinions were grouped into the following categories: rationale for the spread of HIV/AIDS, abstinence, condom use, sexuality and homosexuality, compassion, experiences with PWHA, recommendations and current approach to addressing HIV/AIDS in congregations. Religious representatives expressed a measure of acceptance of HIV/AIDS and overwhelmingly upheld compassion for PWHA. Some statements, however, suggested that HIV/AIDS stigma pervades Trinidad's religious organizations. For many representatives, HIV/AIDS was associated with a promiscuous lifestyle and/or homosexuality. Representatives had varying levels of interaction with PWHA, but personal experiences were positively associated with current involvement in HIV/AIDS initiatives. All 4 PWHA interviewed identified themselves as belonging to Christian denominations. Three out of the 4 PWHA described discriminatory experiences with pastors or congregation members during gatherings for religious services. Nonetheless, PWHA expressed an important role for faith and religion in coping with HIV.

**Conclusion:**

Religious groups in Trinidad are being challenged to promote a clear and consistent response to the HIV/AIDS epidemic; a response that may reflect personal experiences and respect religious doctrine in the context of sex and sexuality. The study suggests that (1) religious leaders could improve their role in the fight against HIV/AIDS with education and sensitization-specifically aimed at dismantling the myths about HIV transmission, and the stereotyping of susceptible sub-populations, and (2) a consultative dialogue between PWHAs and religious leaders is pivotal to a successful faith-based HIV intervention in Trinidad.

## Background

The HIV/AIDS epidemic in the Caribbean region is fuelled by stigma and discrimination, which are the most significant determinants of HIV infection and death from AIDS-related complications [1]. Trinidad and Tobago (T&T), an island nation situated off the coast of Venezuela and home to 1.3 million citizens, [2] shares with region members an incidence of HIV/AIDS second only to sub-Saharan Africa [3]. Since the first case of HIV was identified in Trinidad in 1983 in a homosexual male, [4] the prevalence of HIV/AIDS in adults (15–49 years old) has grown to 2.5%, with half of all new infections occurring in young people between 15–24 years [5]. HIV is primarily transmitted through unprotected sexual intercourse, [6] and is fueled by multiple sexual partnerships, substance abuse, and migration and gender inequalities [7]. The Trinidad and Tobago Ministry of Health suspects the infection is underreported and the actual number of cases is twice as high [5].

Stigma and discrimination create barriers to HIV testing and treatment, care and support networks for people living with and affected by HIV/AIDS [8,9]. In Trinidad, few cases of HIV infection are diagnosed early in the course of infection and the average time from HIV diagnosis to death is only 13 months [4]. Of all confirmed AIDS cases reported in 1999, 75% were identified as HIV positive within the same year as the AIDS diagnosis [4]. The statistics suggest that individuals may fear testing, perhaps as a result of the stigmatization of HIV throughout Trinidad and Tobago's society.

T&T is a spiritually-aware society where the majority of the population belongs to at least 1 of over 104 religious groups [10] that coexist peacefully, often participating in public celebrations of other faiths [11]. When the last census was taken, approximately 30% of the population was Roman Catholic, 24% Hindu, 11% Anglican, 6% Muslim, 3% Presbyterianism and 26% "other" [12]. Religious groups are geographically evenly distributed throughout the nation; active amongst the poorest communities and within areas of high HIV/AIDS prevalence [13]. The pervasiveness of diverse religious ideologies thus creates a complex environment for the transmission of a sexually transmitted infection.

Religious leaders are esteemed, frequently exchange with the public and maintain an influential role in policy-making in Trinidad [11]. They may use their position to promote HIV/AIDS awareness, fight stigma and discrimination in communities, and exercise compassion to facilitate healing for people living with HIV/AIDS (PWHA). Some religious groups are involved in such efforts. In 2001, the Caribbean Conference of Churches (CCC), the Regional Ecumenical Organization of the Caribbean, brought 120 church leaders and church workers from across the region together in a consultation on *"Human Sexuality and HIV/AIDS in the Caribbean – A Theological Approach" *[14]. The consultation raised awareness about the discrimination, fear, rejection, poverty and pain that PWHA may face in Trinidad's society.

Yet there are barriers to more active and widespread involvement in HIV/AIDS initiatives among religious groups in Trinidad. Debate over condom sales, for example, has hindered collaboration with public health organizations [15] and religious groups have not been optimally integrated into the HIV/AIDS care and support network. Further, there are few local research studies that explore religious leaders' incentives to promote and gain involvement in faith-based HIV/AIDS initiatives. One investigation of the potential to inspire a faith-based response to HIV/AIDS in T&T [13] found that HIV/AIDS-related stigma and discrimination inhibited active involvement. The present research expands upon previous work by sampling both diverse religious groups in Trinidad, and individuals who are living with HIV/AIDS. Accordingly, this study investigates how the perception of HIV/AIDS as a sexually transmitted infection impacts religious leaders' incentives to become involved in HIV/AIDS initiatives, and how the experiences of PWHA in religious gatherings have impacted their healing and coping with HIV/AIDS.

## Methods

All relevant ethical procedures associated with collection of interview data were adopted, and the methods approved on behalf of the Caribbean Conference of Churches Ethics Committee. The interviewer explained the nature and purpose of the study to the participants and conducted the interviews in a non-coercive manner, with the assurance of confidentiality. All respondents provided verbal consent, and agreed to be interviewed without financial or other incentive.

### Selection of religious representatives

**Figure 1:**
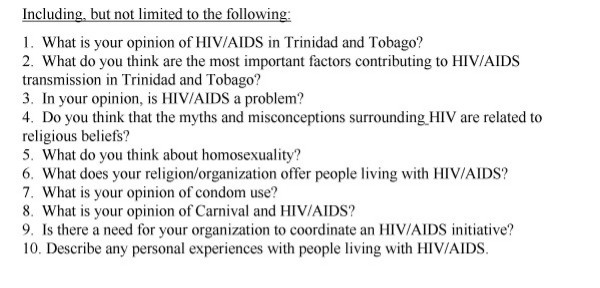
Interview Questions for Religious Representatives.

Between November 2002 through April 2003, in-depth interviews were conducted with 11 consenting representatives from 10 religious organizations in Trinidad, including: Anglican, Open Bible, Pentecostal, Salvation Army, Unity School of Christianity, Seventh Day Adventists, Hindu, Jamaat al Muslimeen, Nation of Islam, and Roman Catholic (2 representatives). Selection of religious groups was based upon the methodology of Brathwaite [13]. Briefly, 26 religious groups with the largest followings according to 2000 census data were identified in Trinidad and Tobago. Groups were categorized into Christian, Hindu, Muslim, and 'native' (Bahai, Orisha). From this original list, 10 representative groups from Christian, Hindu and Muslim denominations with large and small congregations, and located in Northwest Trinidad were selected. Potential participants were contacted by phone, the research plan described, and interviews arranged with willing participants. Religious respondents were generally leaders of religious services (pastors, priests, pundits), while 2 were active congregation members. The majority of interviews with Christian representatives were conducted in offices adjacent to churches. Other interviews were held in an agreed location such as the interviewee's home. Interviews ranged from 1–2.5 hours. Interview questions followed a guideline (see Figure 1), but employed an open-ended question format to invite discussion of sensitive HIV/AIDS-related themes. Those themes were previously identified in a survey of 26 religious organizations, [13] and included: rationale for the spread of HIV/AIDS, abstinence, condom use, sexuality and homosexuality, compassion, experiences with PWHA, and recommendations and current approach to addressing HIV/AIDS in congregations.

### People living with HIV/AIDS (PWHA)

**Figure 2:**
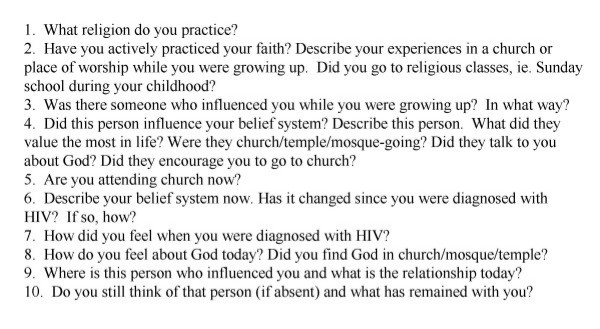
Belief Systems Questionnaire for People Living with HIV/AIDS.

In-depth interviews with PWHA were conducted during the same study period, 2002–2003. Four PWHA (*P1–P4*) were selected from an established HIV/AIDS support group in Port of Spain, Trinidad, based upon their perceived willingness to discuss sensitive issues and religious affiliation. Interview questions were open-ended, but followed a structured guideline (see Figure 2).

### The interviewer

The interviewer was a United States Fulbright Scholar who had studied HIV/AIDS and other public health issues, and intended to use the research on religious groups to stimulate faith-based contributions to ongoing HIV/AIDS initiatives in Trinidad. The interviewer was associated with the HIV/AIDS support group from which the 4 PWHA were selected, and had spent several hours each week interacting with the participants prior to the interviews. All interviews were audio recorded and transcribed by the interviewer.

## Results

Verbatim statements with religious representatives were extracted from raw interview text and were categorized according to the following themes: rationale for the spread of HIV/AIDS, issues related to abstinence, condom use, sexuality, homosexuality, personal compassion and personal experiences with PWHAs. Similarly, the responses of PWHA were grouped into three main themes: response to HIV diagnosis, experiences in church, and current attitude toward religion.

### Introduction to religious groups

#### Anglican Church (AC)

In keeping with *"a more collegial approach to ministry," *the Anglican community was engaged in educational programs, such as peer counseling, to sensitize and educate the clergy on issues surrounding HIV/AIDS. According to the *AC *representative, *"AIDS is one of our chief priorities: our thesis that the faith based communities must be part of the solution than a problem by being negative and adversarial versus pastoral...."*

#### Open Bible

The respondent felt that because HIV/AIDS prevalence in T&T had not reached critical proportions like in Africa, it was not an urgent problem. There was no written policy on HIV/AIDS because *"we have not had a lot of cases to deal with. I guess if we had that amount we would. We have treated every case on an individual basis." *It was felt that if the problem were addressed on a national level, churches would necessarily unite to develop a joint initiative.

#### Pentecostal

The representative upheld compassion for all individuals, recognizing the dignity of every human life. The organization would assist PWHA in order to help to facilitate conversion and lifestyle changes that would lead to an improved contribution to the community. HIV/AIDS has caused a devastation that requires communities and individuals to reexamine and change their behavior.

#### Roman Catholic (RCI, and RCII)

*RCI *believed the degradation of the traditional family unit, the erosion of traditional cultural values and undefined rites of passage have contributed to the spread of HIV/AIDS. Religious instruction in the Church has failed to meet the present day needs and concerns of the congregation. *RCII *felt that HIV/AIDS must evoke a discussion of sexuality and of how individuals could prepare themselves to make informed, meaningful and healthy choices that reflect who they are and not simply what they want. To date, the Roman Catholic Church has very effectively delivered home health care to individuals suffering from AIDS-related complications through ministries such as Caritas.

#### Salvation Army (SA)

The Salvation Army was founded in 1878 by William Boothe, a Methodist minister in England, who wanted to help the poor of England receive food, shelter and clothing. Thus, the organization is based on a philosophy of outreach, serving community needs, helping the sick and the poor. *SA *"does not *condone sin...but we love the sinner*," and would work with individuals to build a better lifestyle. *SA *advocated chastity, but condom use was preferred to acquiring HIV for individuals who choose to engage in sexual intercourse before marriage.

#### Seventh Day Adventist (SDA)

There was a written policy on HIV/AIDS, but the interviewee was not familiar with the text in the policies. The interviewee was disappointed in the rate at which the SDA organization had responded to the HIV/AIDS epidemic in Trinidad and acknowledged that there was the need for further education of clergymen within the church. Increasing education in society and among the clergy was the most critical component of reducing the spread of the virus.

#### Unity School

Unity was a small congregation of mostly women, who were described as traditional and conformist. HIV/AIDS did not affect the congregation and the representative did not have personal relationships or experiences with PWHA. The organization was not involved in outreach. Unity is based upon the philosophy that "*human beings create their experiences by the activity of their thinking*." Therefore prayer is "*creative thinking that heightens the connection with God*."

#### Hindu

The *Hindu *representative felt that HIV/AIDS occurred primarily among homosexuals and did not pose a significant problem for the Hindu organization. Furthermore, it was assumed that Hindus were less likely to acquire HIV due to high social and spiritual obligations to obey religious doctrine. According to the interviewee, HIV/AIDS was a medical problem. Although prayers and mantras are effective treatments and cures for disease, the organization was less concerned about bodily ailments than it is about eternal life. The interviewee felt that individuals living with HIV/AIDS in the Hindu community may feel discriminated against and ostracized because disease is an *"unhygienic situation;" *individuals living with HIV/AIDS are unclean and would be expected to stay away from organized worship. Compassion is inherent in Hinduism, but the religion does not provide the opportunity for confession and reconciliation.

#### Nation of Islam (NOI)

*NOI *supported a theory that HIV was man-made in a United States laboratory in a plan to control population growth. The *NOI *representative was skeptical of scientific literature and research on the efficacy of condoms and boldly supported abstinence as the only effective prevention of HIV and other STDs. *NOI *valued the individual, and is particularly protective of women. The organization fulfills the obligation to help anyone in need.

#### Jamaat al Muslimeen

This representative was highly active in HIV/AIDS education for the neediest urban communities. *Jamaat al Muslimeen *traveled on foot to reach individuals and families who demonstrated serious health risks, and provided education and condoms. However, the representative's activism, particularly in condom distribution, was not supported by the Jamaat al Muslimeen organization. According to the interviewee, the organization believed HIV/AIDS was a *"sin from God."*

### Rationale for the spread of HIV/AIDS

Religious representatives described cultural, spiritual, and social factors that contributed to the spread of HIV/AIDS. *RCI *believed that new opportunities for HIV transmission were born in the fragmentation of traditional family structure and the erosion of the influence of religious doctrine in society. According to the *Pentecostal *representative, *"if you follow the pattern that is established here in the Word, that you are not going to get into trouble, or there will be no involvement in sexual accounts...like HIV...or any of the other kinds...." *The *Open Bible *representative *"really believe that this started with homosexuality. They can't find a cure for it...what you sow, you reap."*

The "Carnival mentality," was also felt to fuel the epidemic. Group representatives generally described how Carnival encouraged individuals to abandon their moral framework. The *Hindu *representative did not feel that Hindus would engage in this behavior, however.

Pentecostal: "It's what people are making of the Carnival mentality. If people would enjoy themselves and see Carnival as functional in terms of your socioeconomic...we will not have this kind of [situation]... [People] let their standards down...to do what they would not normally do. And this to me is prostituting Carnival...To go around and have sex, as if, you know, you have no control."

Open Bible: "Carnival is one of the greatest contributors to AIDS...there is no limits, there is no restrictions. People just let down all there guards that they will have had all year...And only afterwards they realize they make mistakes...too late."

Hindu: "You find too that around Carnival time...this is highlighted most of all, and you would find that many Hindus would not be seen in the streets taking part in Carnival. You may see [East] Indians, people of Indian descent, many of them would have already crossed over to other religions. And so the guard is already dropped...promiscuity, and licentiousness...."

### Abstinence

There was little variability among religious groups in response to the organization's position on abstinence.

Pentecostal: "We are not going to tolerate at all any sexual activity outside of the bonds of marriage. We go in accordance with what the Word says that marriage is honorable in all, and the bed undefiled-the only time the bed is undefiled is when there is marriage."

Salvation Army: "Sex before marriage is not acceptable in the Christian Church."

SDA: "I believe that we are aware of the reality, of not everybody will abstain, but we would emphasize abstinence, from a doctrinal point of view, and because we believe that it is the safest... Our thing of abstinence is not strictly about avoiding HIV; abstinence is also about...pregnancy, it's about...sexual relationships, where you have amount of responsibility...If it's with somebody you are very interested in...not just a one night stand."

NOI: "...the sole objective is to promote abstinence-not the fact that you can get an STD. I don't relate to that at all. And we don't go around, you know, promoting condom use, we promote abstinence because there is a much greater value attached to the individual, particularly the female that if she was sensitized and made aware of that and I think that would be much more effective for her than the condom."

### Condom use

Within Christian denominations and across religious groups, positions on condom use differed, ranging from an acceptance of condoms in lieu of the "reality" of HIV/AIDS, to a general contempt for their use as a substitute for self-control.

SDA: "From my personal point of view, you cannot promote the use of condoms, because in promoting the use of condoms, what you're actually doing is telling the person you cannot control your sexual urge. So because you cannot control your sexual urge, here is something to use when the urge comes...And you find that as a country growing, so many young people are contracting HIV in spite of the availability of condoms."

RCII: "What I was very clear about myself was that...we are not talking about condoms as contraceptives when you talk HIV. That the church's condemnation of condoms is about contraception; you are talking about a contra-abortive, which is people dying."

*Pentecostal*: *"We don't think that it is fair or right to distribute condoms to... like young people who are not married. You are not supposed to be actively involved with sex outside of marriage, so any person or persons outside of marriage, we feel, should not really have the use of any condom. Condoms within the marriage we feel, should, in our view, be a matter for the people involved."*

Salvation Army: "Sex before marriage is not acceptable in the Christian Church, but being practical in this day and age, better use a condom than get a disease."

Open Bible: "...teaching the people the importance of not being promiscuous...providing condoms...that doesn't fix the problem...the problem is the individual...so start from small...there is a whole lifestyle that starts from a little child, and you have to start there."

NOI: "I read one interesting quotation from a medical professional that has stayed with me. And it says that the AIDS virus passes through...the membrane of a condom like a golfball passes through a basketball hoop...."

The *Jamaat al Muslimeen *organization forbids condom use. However, the interviewee's position was in marked contrast to that held by the organization. The *Jamaat al Muslimeen *representative carried condoms on foot to the poorest of Trinidad's communities in opposition to the philosophy of the organization:

Jamaat al Muslimeen: "And what I do also is that I distribute condoms. And a lot of people that I have spoken to in the underdeveloped community, most of them use condoms sometimes, and sometimes they don't use condoms... we try to educate them towards the disease...."

### Sexuality and homosexuality

Adherence to religious doctrine and the fear of shame are thought of as protective factors that insulate the *Hindu *group from a promiscuous lifestyle. The *Unity *group felt that Trinidad and Tobago was *"probably the most promiscuous little country in the world." *Roman Catholic representatives were concerned about the growing number of young children voluntarily initiating sexual activity, and believed that sex among the youth was becoming a defining characteristic of the culture. The *RCII *representative explained that gender equality provides enormous potential for mutuality, and needs to be explored by society.

RCII: "Sex is a powerful potent force in human society.... For me the one thing that is difficult in Caribbean society that is distinct from some more traditional societies, is that the kind of rituals of initiation which have allowed people to claim manhood, womanhood without become sexually active in the open sense, those rituals don't exist.... Sexuality is extremely fragile...we talk about it simply as something that we do when in fact it is something that you are...."

According to many representatives, education on sexuality in the context of religious doctrine was unnecessary, because religious tenets sufficiently define appropriate behavior: if one upheld the teachings in the sacred texts, HIV/AIDS would not be transmitted or acquired. The opinion on homosexuality was generally uniform across religious denominations, although personal attitudes varied in their degree of outrage; some groups called it *"abominable," *or *"sickening"*. Some interviewees, however, expressed the potential for homosexuals to be converted, and adopt an acceptable form of behavior.

Open Bible: "We are not against homosexuals but we don't promote homosexuality...we strongly disagree with it. We believe that God never intended for people to live in a homosexual relationship and so certainly we don't in any form or fashion, entertain it...."

Unity: "AIDS is not new. I don't think AIDS comes from any homosexual behavior. I think people are capable of loving."

SDA: "From my personal observation I don't think that homosexuality is something that our ministers have a lot to do with...they have not done a lot of interacting."

Hindu: "In all religions, sex is looked at as very sacred. Whereas you would not find the Hindu woman covered all over like the Muslim, they ought to be quite protected, and I think from the woman folk point of view, it is even less a threat. You would find that homosexuals mainly from the male contingency, and...he would be out there in the world, and he may encounter certain situations, and he may get into...this promiscuous activity and may become homosexual...."

### Compassion and religious representatives' experiences with PWHA

Personal interactions between group representatives and PWHA varied widely. Some representatives had buried individuals who had died from AIDS-related complications. The *Jamaat al Muslimeen *representative personally contacted individuals and communities in Port of Spain affected by HIV/AIDS to provide them with condoms, support and education. In contrast, other representatives, including *Open Bible*, *Unity*, and *Hindu*, reported little to no personal interaction with PWHA. Nonetheless, religious groups unanimously supported compassion for people living with and affected by HIV/AIDS. The expression of compassion was often associated with conversion and a desire to *"help the person change."*

RCII: "The point of breakthrough was to equate HIV positive persons with lepers in the Gospel story, so Jesus came for the lepers, he's come for them. Which is so horrible...but it was a way for the churches to open up to it...

"There was a conversion process involved in many people... from AIDS as punishment from God to AIDS as a sad event in human history which now demands a response from those who say they believe in the name of God, but that that response must always be compassion...."

Anglican: "Last month I buried at least two persons with AIDS. A twenty-five year old male, thirty-eight year old female-she sold drugs and she also sold herself-her body. A sad, sad, sad moment...AIDS is not an academic thing here...it is very concrete...."

Pentecostal: "So we feel we need to relate with them treat with them as members like anyone else and we ought to show that the same kind of love, the same kind of respect...and we do that."

The *Pentecostal *representative also articulated how myths about HIV transmission were dispelled in personal interactions with PWHA:

Pentecostal: "Many of the myths are...HIV can be spread with the use of utensils-many believe in those myths even within the church...The fallacies...or the myths have been put to rest for us...because of the experiences we have had dealing with people who have had HIV...."

Salvation Army: "We actually had an HIV/AIDS person who was very close to us in our formal apartment...he was bold enough to tell us he had the problem, and so we helped him a great lot...we knew that he needed extra food...he was rejected from his own house, and we had to help him get settled...so we sort of have an idea...it is a problem that needs help. If you come to me and say I have HIV/AIDS, I am not going to say that you did something wrong... We have reached out into many areas of social work because we have a heart for people."

SDA: "The churches responsibility is to show compassion. And not to check and find out how this person managed to get it. I think most Adventist churches are moving away from the medical fact...."

Hindu: "This religion is based upon compassion, if you do not have compassion, you are lacking in one of the major ingredients to be a Hindu. So we do not go about branding anybody, saying okay, you are a sinner. It is more understanding, it is also a recognition of the unhygienic situation that arises."

Jamaat al Muslimeen: "I have a twenty-one year old girl, she's HIV positive...I was even trying to get her to get public assistance because she and the two children are HIV positive. And I really thought that she was trying to turn around her life. But she just kept going with men without condoms."

According to some religious representatives, coping with HIV/AIDS was different than coping with other terminal diseases that are not sexually transmitted, such as cancer: people living with HIV/AIDS come to church requesting confession, whereas people fighting cancer want to be healed. The *RCII *represented expressed the contrast this way:

RCII: "What she was showing was a different form from someone who has cancer. But the fear that I saw in her was different from the fear I saw in cancer patients. It was fear coupled with guilt, and of course she came asking me to pray plenty... you hear the AIDS people telling you that there's a certain anger with themselves."

### Recommendations and approach to addressing HIV/AIDS in congregations

Religious organizations differed on how to confront the HIV/AIDS epidemic in Trinidad, if at all. Some representatives, like *RCII*, began their involvement in HIV/AIDS care and support networks early in the epidemic, while others remained insulated.

RCII: "But what they did very well was to train a number of people would could go into homes and provide home care for people who had nobody else to care for them...More recently, the schools have been involved in education and the Catholic Church is in the process of providing, producing a video about HIV."

Open Bible: "I don't think there is a need to have something structured in place. I guess in Trinidad it is still a very private matter...I don't think that this can be addressed by any one local church, I think this is something that is more a national issue...and if it has to be dealt with then churches have to get together to deal with it because of the scope of it."

Anglican: "If you don't arrest this AIDS thing...what will happen is that so much money will be spent on AIDS and people infected with the virus, you have little or no money to spend on cancer, and diabetes, and all the other things."

Pentecostal: "Obey the word, abstain from sex, and avoid HIV."

SDA: "We have been more or less targeting young people. A lot of workshops going on as well. We run a home care training workshop, we also do a sensitizing program for our young people...we try to bring across in as many of our programs as we can...activities that relate to HIV and AIDS, so that we are educating our young people. In terms of our ministerial staf...there are courses that they must necessarily do in the program... there is a health course that ministers must do while they are there in their training, and that of course exposes them to the myths and realities of HIV and AIDS...."

Hindu: "When I started thinking more deeply, I thought to myself, this is not so much of a Hindu perspective, or a Christian perspective, or a religious perspective, but a medical issue...I think that the avenue provided for help, is one of a spiritual environment. But it is not through religious bodies."

### Religion in the lives of PWHA

Both Roman Catholic representatives provided a rationale for seeking God to facilitate coping with an HIV diagnosis:

RCI: "Well, they're in a hopeless situation! They're beyond human help and so the next thing you turn toward God."

RCII: "Because this is fundamentally a religious society. If you say you don't believe in God, doors close in your face. If you say you believe in God you stand a much better chance of getting help from certain quarters. Plus the psychology of HIV, you come face to face with your own mortality. Turning to God is probably the most natural thing to do...Once you enter into a mode where you think you're dying you go through a whole process of anger, of bargaining."

Four PWHA described their experiences in church and their spiritual journey subsequent to receiving an HIV diagnosis. One respondent agreed with religious representative, *RC1*, in that HIV was a *"crisis" *situation. Managing feelings of guilt were an important part of the initial coping process. When 1 of the interviewees was diagnosed with HIV, she felt she was being punished for committing a sin, and her pastor confirmed those feelings. All 4 individuals described discriminatory experiences by clergy or congregation members, but for 3 of the 4 PWHA, negative experiences did not affect attendance nor diminish a spiritual journey. HIV diagnosis generally inspired a desire to explore spirituality.

The first interviewee, *P1*, went to church irregularly growing up. When she was diagnosed with HIV in her early twenties, the church provided peace and solitude during her *"crisis." *However, she stopped attending when she suspected her pastor ostracized PWHA.

#### Response to HIV diagnosis

P1: "With HIV that I went. Before I never really had any kind of crisis. If a boyfriend and I split up, that wasn't a crisis; they had other guys out there. It was really when I found out about my status...I would go to church and people would see me crying...crying down the place...just tears. Letting my heart pour out and talking to God."

#### Experiences in church

P1: "I went to the church for solitude to get some sense of peace some kind of understanding as to why this is happening to me...we decided that I could speak to the pastor's wife. And she sat down with me and she said, you know she had a son who also died from it. I mean nobody would have expected a pastor's son to get HIV because they're not supposed to be living a promiscuous life...She comforted me and she told me you know I'm welcomed in this church at anytime.

I came and I told [my friend] about it. She knew the guy who died, her son. His family was not nice to him. His mother his father was not nice to him. For her to be giving me another story. It was unbelievable to think that they were not nice... I think that is one of the reasons too that I did not go back."

#### Current attitude towards religion

P1: "I kind of gave up on myself and I started going to a lot of parties. So when Sunday came, I would go party Saturday night and when Sunday came I can't get up in the morning to go to church. And that kind of threw me off from going, from attending that. But I want to start back going."

*P2 *grew up attending church daily with his mom, who was Roman Catholic. Later in life he experimented with drugs and sex. Near-death experiences were the impetus to reach out to a spiritual counselor, and he later became active in community outreach programs.

#### Response to HIV diagnosis

P2: "I was thinking about dying all the time. The addicts felt sorry for me.

But I don't feel like dying anymore. It is only by the grace of God that I am not depressed. What I care about doing the right thing, taking my medication and learning more about it. I would like to carry the message to schools ...I don't feel any less than anybody because I know that Jesus Christ loves me and I trust him."

#### Experiences in church

P2: "I talked to the pastor and a few of the deacons high in the Church and when I was sick they didn't come by. I felt they didn't respond as they should have. Last Saturday my pastor shook my hand."

#### Current attitude towards religion

P2: "God is my armor, my weapon has made me overcome a lot of hurdles and a lot of hang-ups...it has played a very important part...even with my addiction my spirituality has changed a lot. I choose God."

*P3 *was active in an SDA church, but before her HIV diagnosis was a member of an Islamic organization.

#### Response to HIV diagnosis

P3: "It happened in 1995...and I couldn't believe that I was HIV positive, because at that time I was not educated about living healthy with HIV. So I thought to myself that it would be the end of the world for me.

...I decided, listen if I have to die I need to make peace with my God, whoever the creator is...I also had a friend, who was a Christian...and he said to me Christ could help you, Christ can heal you...I started reading the Bible, with a longing in my heart to find out...if the creator was hearing me or not. I used to pray like five times a day because my body was diminishing, my hair was falling off, I had sores all over my body...things just started getting clearer and clearer to me, and my eyes were just opened, slowly but continually being opened to what is real, what is the reality of life."

#### Experiences in church

P3: "I was impressed to let the church know of the power of God, because I know it was a miracle, and I wanted them to know that the God that they serve is still in the business of doing miracles...But they did not respond to it very well, therefore I was faced with plenty stigma, and word got around...and I just saw everybody start whispering...Now I'm faced with a reality...if I get involved with anybody, everybody...scorn me, and any young man within the church comes around me, bet your bottom dollar someone's going to tell them, she's HIV positive."

#### Current attitude towards religion

P3: "It's all well and fine that governments are looking into HIV care and treatment, but why isn't the religious sector taking part in a more meaningful way? Presently I am trying to get the Adventist, we already we have an AIDS ministry, but they are not involved in a holistic view in terms of educating even their own people, far less the general public.

The church has to play a very important role in the fight against HIV and AIDS... The reason that I think that the virus has mushroomed, is because of moral standards...moral standards have gone down."

*P4 *regularly attended and was active in a Pentecostal church. The HIV diagnosis was confirmed at 18 years old.

#### Response to HIV diagnosis

P4: "I thought I was being punished for my sins. That's what he said too. I am being punished. And I caused it on myself, and on and on and on he went. But I don't think that is true...The first camp I went to after I was diagnosed, I talked to a social worker. She used to really encourage me. The first time I went to her I was really depressed, and asked her if she thought God was punishing me for my sins. She said, is God evil? What about all the children born with HIV, are they being punished? Bad things happen to everybody."

#### Experiences in church

P4: "I talk to my pastor. But I come to find out that he was not so trustworthy...I went to my pastor and I was talking to him about it and I find out that he was telling everybody...He said they needed to know so that they could pray, so the church could pray. But he just said that so he could tell everybody...Once the board knows-the board is family member leaders. I think everybody probably knew or was told by someone else. I still see him. I'll say hi. That was about a year and a half ago.

The last time I went to church I was talking to this little boy, and this woman told me not to touch him, if you touch him you will give him your germs. The boy was six months old. I learned a lot."

#### Current attitude towards religion

P4: "Despite all the real bad things, I believe in my religion. I guess in every organization you'll find good people and bad people...I'm always really love my religion, love church and love God. To myself I feel comfortable, so I don't think people should influence my relationship with God, I'm really, really, really trying to work on it."

## Conclusion

Christian, Hindu and Muslim religious representatives differed in their attitudes and opinions on the following themes: rationale for the spread of the HIV/AIDS epidemic in Trinidad, sexuality and homosexuality, condom use, processes for healing, and the impetus to become involved in faith-based initiatives. Levels of awareness about the prevalence of HIV/AIDS in Trinidad, susceptible sub-populations, and knowledge of the mechanisms of HIV transmission also varied. Religious representatives isolated subgroups who were believed to be particularly susceptible to HIV infection, and in so doing, implicitly articulated HIV/AIDS stigma in different ways. For example, the *Hindu *representative believed that HIV infection was generally limited to homosexuals and promiscuous non-Hindus. For the *SDA *and *Pentecostal *representatives, HIV was synonymous with a promiscuous lifestyle and the transgression of abstinence.

Representatives were generally in agreement in their advocacy for the sanctity of marriage prior to engaging in sexual intercourse. In correlation, the *SDA *representative felt that condoms facilitated the transgression of abstinence and the degradation of self-discipline. The *NOI *representative felt that condoms aided and abetted promiscuity in society, but went further in claiming that condoms were not even an effective barrier against sexually transmitted infections. In contrast, the *"reality" *of HIV/AIDS for the *SA *representative led to a more accepting attitude toward condom use. The *Jamaat al Muslimeen *and *RCII *representatives had personal experiences with PWHA, and it was their understanding that condoms were life-saving tools. Thus, the present research also suggested that personal experiences and interactions among religious representatives and PWHA dispelled myths surrounding HIV/AIDS transmission, and sensitized individuals to the HIV/AIDS *"reality." *The *SDA *interviewee believed that personal experiences with PWHA were critical to the HIV/AIDS sensitization process, and for dismantling myths about transmission.

The full potential for religious groups to contribute to HIV/AIDS awareness efforts is currently untapped. While the majority of representatives admit that HIV/AIDS is a serious problem that is affecting the country and the world, there was wide disparity in the impetus for implementing a faith-based initiative targeting HIV/AIDS-related issues. The *Anglican *interviewee supported a proactive initiative and HIV/AIDS was among the Church's 5 priorities; however for the *Pentecostal*, *SDA*, *Open Bible *and *Hindu *groups, HIV/AIDS was not a priority that needed immediate attention and warranted discussion among congregations. According to the *Pentecostal *representative, HIV could simply be avoided by adhering to the behavioral conduct outlined by Christian tenets. Despite the *Jamaat al Muslimeen *representative's personal efforts in raising awareness about HIV/AIDS in rural communities, she felt that the "mix" of HIV/AIDS initiatives and faith-based communities invited stigmatization of PWHA.

Whether it was called *"divine purpose" *according to the *Hindu *representative, *"openness to the transcendent," *by the *RCII *interviewee, or the *"God conscious part" *by the *Open Bible *representative, it was agreed that humankind are inherently spiritual beings; and that Trinidad is indeed a spiritually-aware society. For the 4 participants living with HIV/AIDS, all of whom identified themselves as Christians, an HIV diagnosis inspired an exploration of spirituality, and led to a deeper connection with God-despite experiences of isolation and discrimination in church. One PWHA was identified as HIV positive by her pastor during a worship service, so that he could exemplify deserving consequences of sexual behavior. The attitude of this pastor seems to reflect the opinion of the *Open Bible *and *Pentecostal *representatives: by abstaining from sex one avoids HIV. Other PWHA were also discriminated against by members of the congregation. Nonetheless, for 3 of the 4 individuals, negative and discriminatory experiences did not affect attendance in church nor attenuate a spiritual journey.

Religious representatives were generally willing to participate in a care-giving capacity for people with HIV/AIDS because these efforts were built into their existing mission. Religious groups in the past have publicly acknowledged a responsibility and desire to be involved in care-giving for people living with and affected by HIV/AIDS. For example, organizations such as the Roman Catholic-sponsored Caritas have been successful in home health care for PWHA and their families. At the 1998 Youth Summit, religious representatives formulated "support resolutions," recognizing the need for their involvement in communities through the provision of information and counseling services for adolescents in society [16]. A 2001 report revealed that religious groups desired to improve their capacity to contribute to care and support [13].

Regional conferences such as those led by the CCC indicate that religious groups are beginning to mobilize in confronting the HIV/AIDS epidemic. Since the completion of this study, in 2005, the CCC hosted another consultation with Faith Based Organizations to develop an HIV/AIDS policy and action plan, entitled, *"Guidelines for Caribbean Faith-Based Organizations in Developing Policies and Action Plans to deal with HIV/AIDS" *[17]. The document is part of a collaborative effort, *"Building a Faith Based Response to HIV/AIDS in the Caribbean" *to enhance the response of faith-based organizations to Trinidad's HIV/AIDS epidemic. Furthermore, the highest level of government supports the crucial role religious groups may play in mitigating the impact of HIV/AIDS stigma and discrimination. National HIV/AIDS prevention efforts involving faith-based organizations are mandated by the 5 year HIV/AIDS National Strategic Plan (NSP), whose goals include the provision of necessary support within a holistic framework for those persons infected and affected by HIV/AIDS. These goals are currently undertaken by the newly-formed National AIDS Coordinating Committee [18].

Nonetheless, several religious representatives agreed that the pace of efforts on behalf of religious organizations has been too slow. The present research raises the following question: if HIV were not sexually transmitted, would religious organizations respond to the epidemic in the same way? The HIV/AIDS epidemic in Trinidad urgently calls upon religious groups to provide a clear and consistent response to issues of sex and sexuality that resonates with personal experience and respects religious doctrine. However, the present research highlights inconsistent attitudes and opinions on the moral and spiritual issues surrounding HIV/AIDS; such inconsistencies may serve as a barrier to a united faith-based initiative in Trinidad. Religious groups across all faiths and denominations are challenged to recognize that human beings are sexual beings; herein lays the dilemma for religious groups. A faith-based intervention must understand the complexity of preserving the central tenets of organized religion while embodying compassion for individuals as sexual beings.

This study was limited by a small sample size and the geographic location of Northwest Trinidad; this may affect the generalization of results throughout Trinidad and Tobago. On average, only one representative from each religious organization was interviewed, and opinions expressed did not necessarily reflect those of the religious organization as a whole. Nonetheless, HIV/AIDS-related stigma and discrimination will continue to fester throughout Trinidad and Tobago until all the republic's religious leaders, esteemed in the public eye, possess accurate information about HIV transmission, which may then be conveyed to congregations; and until religious leaders are sensitized to the experiences of PWHA. Prior to involvement in community-based education, care and HIV/AIDS support initiatives, religious leaders must possess compassion that is reinforced by personal experiences with PWHA. PWHA are receptive to faith-based counseling and support provided by religious leaders and congregation members. Thus, a consultative dialogue between PWHA and religious leaders is pivotal to a successful faith-based HIV/AIDS initiative in Trinidad.

## Abbreviations

**AC: **Anglican Church

**AIDS: **Acquired Immuno-Deficiency Syndrome

**CAREC: **Caribbean Epidemiology Center

**HIV: **Human Immunodeficiency Virus

**NOI: **Nation of Islam

**PWHA: **People Living with HIV/AIDS

**RC: **Roman Catholic (group representatives, *RCI *and *RCII*)

**SA: **Salvation Army

**SDA: **Seventh Day Adventist

**STI: **Sexually Transmitted Infection

## Competing interests

The findings and conclusions in this report are those of Dr. Brathwaite and Ms. Genrich and do not necessarily represent the views of the funding agency, the Institute for International Education and Fulbright Fellowship Program, nor the organizations where the authors currently work, the University of the West Indies and the Centers for Disease Control and Prevention, respectively. There were no competing interests, financial or otherwise, in the present investigation, and no incentive on behalf of the Institute for International Education to obtain the results found. No fees or funding were obtained from any organization that could gain or lose from the publication of the manuscript, and no stocks or shares are held in any organization that stood to gain or lose financially from publication.

## Authors' contributions

Dr. Brathwaite contributed to the design of the research method. Ms. Genrich carried out the in-depth interviews with the participants, religious representatives and individuals living with HIV/AIDS. Both Dr. Brathwaite and Ms. Genrich collaborated on the final analysis and manuscript preparation. Both authors read and approved the final manuscript.

## Pre-publication history

The pre-publication history for this paper can be accessed here:


